# The immunobiology of SARS-CoV-2 infection and vaccine responses: potential influences of cross-reactive memory responses and aging on efficacy and off-target effects

**DOI:** 10.3389/fimmu.2024.1345499

**Published:** 2024-02-26

**Authors:** Craig P. Collins, Dan L. Longo, William J. Murphy

**Affiliations:** ^1^ Graduate Program in Immunology, University of California (UC) Davis, Davis, CA, United States; ^2^ Harvard Medical School, Brigham and Women’s Hospital, Boston, MA, United States; ^3^ Departments of Dermatology and Internal Medicine (Hematology/Oncology), University of California (UC) Davis School of Medicine, Sacramento, CA, United States

**Keywords:** immunology, vaccination, SARS-CoV-2, anti-idiotype antibodies, aging

## Abstract

Immune responses to both SARS-CoV-2 infection and its associated vaccines have been highly variable within the general population. The increasing evidence of long-lasting symptoms after resolution of infection, called post-acute sequelae of COVID-19 (PASC) or “Long COVID,” suggests that immune-mediated mechanisms are at play. Closely related endemic common human coronaviruses (hCoV) can induce pre-existing and potentially cross-reactive immunity, which can then affect primary SARS-CoV-2 infection, as well as vaccination responses. The influence of pre-existing immunity from these hCoVs, as well as responses generated from original CoV2 strains or vaccines on the development of new high-affinity responses to CoV2 antigenic viral variants, needs to be better understood given the need for continuous vaccine adaptation and application in the population. Due in part to thymic involution, normal aging is associated with reduced naïve T cell compartments and impaired primary antigen responsiveness, resulting in a reliance on the pre-existing cross-reactive memory cell pool which may be of lower affinity, restricted in diversity, or of shorter duration. These effects can also be mediated by the presence of down-regulatory anti-idiotype responses which also increase in aging. Given the tremendous heterogeneity of clinical data, utilization of preclinical models offers the greatest ability to assess immune responses under a controlled setting. These models should now involve prior antigen/viral exposure combined with incorporation of modifying factors such as age on immune responses and effects. This will also allow for mechanistic dissection and understanding of the different immune pathways involved in both SARS-CoV-2 pathogen and potential vaccine responses over time and how pre-existing memory responses, including potential anti-idiotype responses, can affect efficacy as well as potential off-target effects in different tissues as well as modeling PASC.

## Introduction: the diverse immunology underlying SARS-CoV-2 and vaccine responses

The ongoing SARS-CoV-2 (CoV2) pandemic, with its devastating health and economic effects, has generated an urgent need to gain more in-depth understanding of the complex and interdependent immune mechanisms at work in response to both the virus and to the vaccines that have been developed to combat it. This has been particularly important given the continual emergence of new viral variants which increase in their immune evasive properties, resulting in the need for further vaccine optimization and periodic application. This need has also been highlighted by the extreme diversity of immunological effects observed within the population after CoV2 infection (and reinfection) or vaccination. At one end of the spectrum, some infected patients can have over-reactive immune responses resulting in a life-threatening pro-inflammatory cytokine storm, necessitating the need for immune suppression (1). Aging and obesity appear be risk modifying factors on pathogenesis and outcome. Conversely, many younger individuals can present with relatively asymptomatic infections with rapid viral clearance and spontaneous resolution (1, 2). Similarly, immediate adverse immune reactions to the various vaccines in otherwise healthy adults, while rarer, are also diverse, with some developing rapid allergic reactions and some, even less frequently, with potentially serious off-target effects such as myocarditis and thrombotic events (3). It is still unclear as to what effects are due to vaccine application versus possible past or concurrent CoV2 exposure, especially given recent data indicating that both CoV2 and S protein can be detected in some patients long after viral infection resolution (4, 5). Finally, a growing body of data accrued from patients has shown that various symptoms can persist for many months after infection, called post-acute sequelae of COVID-19 (PASC) or, more colloquially, “Long COVID,” indicate that immune-mediated pathways are involved.

Regarding the potential of vaccine-mediated effects, some of these immune-mediated events may be intrinsic for the type and construct of vaccine applied (mRNA, adenovirus, inactivated virus, protein or protein fragment, use of carriers like polyethylene glycol, etc.), as well potential effects of the antigen targeted (i.e., the spike (S) protein in CoV2 which can have direct proinflammatory effects (6)). The immune responses induced to both the virus and vaccines likely play a driving role as well. The occurrence rate of these adverse effects are also notable and being increasingly appreciated as more data are generated. A recent report detailing that 70.79% of those that who were vaccinated and participated in a questionnaire had experienced side effects after the second dose of vaccine, while 46.76% of participants that had already experienced infection had adverse effects after the first injection (7). Another study observed that elderly patients experienced adverse events at a higher rate than other groups, with tachycardia, hypertension, and hypotension being commonly reported, though more serious events, like acute myocardial infarction and cardiac arrest, were also reported (8). Long-term effects of CoV2 infection have also been reported, with men being at an increased risk for cardiovascular complications after CoV2 infection (9, 10). Others have also reported similarly increased occurrence rates of adverse events, at elevated intensity, in the elderly population with repeated vaccination, with those having infection prior to vaccination typically having elevated rates of occurance (11). In comparison to adverse occurrence rates in the elderly with other common vaccinations (influenza, Td, Hepatitis B, etc.), adverse events have been reported to be lower than that observed with SARS-CoV-2 vaccination, though a direct comparison study has not yet been performed (12, 13). In addition, others have reported that mRNA SARS-CoV-2 vaccines have been associated with a higher occurrence of adverse events in comparison to adenoviral vector and inactivated virus vaccines (14, 15).

The diverse array of long-term and diverse effects defining PASC implicate multiple organ systems (cardiovascular, pulmonary, neurologic) being affected, similar to those reported with primary CoV2 infection itself (16). Data supporting the implication of the immune system mediating, at least in part, the development of PASC, is compelling, even considering the heterogeneity of immune responses reported. Primary infection has been demonstrated to cause apoptosis to hippocampal cells, as well as alteration of the neuronal landscape and cognitive impairment (17, 18), with preclinical studies also indicating neuronal inflammation even after clearance of the virus (19). Spike protein has been reported several months post-infection as well (5), with data demonstrating that the S protein has the ability to cross the blood brain barrier and cause inflammation through TLR triggering and inflammasome activation (20–22). While reports of possible long-term effects following vaccination have been shown (23), attributing these solely due to the vaccines is extremely problematic, given the reliance on clinical data and history. The presence of concurrent viral infections, such as asymptomatic CoV2, or common latent infections like Epstein-Barr virus (EBV) and cytomegalovirus (CMV), or continuous exposure to endemic hCoVs, can all be affecting immune responses and the symptoms reported. Additionally, the frequent emergence of CoV2 variants continuously alters immunologic epitopes targeted, which has further complicated the picture newer vaccine formulations are produced to combat them. Some of the initial data suggesting that pre-existing immune responses may affect CoV2 immunity revolved around the reported relatively rapid waning of protective antibody responses (within months), as well as reported reinfection rates (24, 25). More recent data has also demonstrated with both viral infection and vaccination that class switching of antibodies to IgG4 from IgG3 and IgG1 occurs, with IgG4 being a lower affinity subclass of IgG, compounding the issue of total antibody levels waning (26–28).

Given the tremendous heterogeneity of immune responses, pleiotropic pathologies reported, and the emergence of PASC, how can true mechanistic studies be performed to delineate not only causation but also treatment options? Unfortunately, most data regarding both CoV2 infection and vaccines have relied on imprecise measures of human immune function (primarily serum antibody levels or cytokines), variables known to be influenced by a number of clinical factors including underlying medical conditions, and to reflect *in vivo* immune function imperfectly as well as being highly variable and affected by numerous factors. It is only through the use of preclinical studies that controlled situations and experimental conditions can occur that delineate these different questions.

## Preclinical insights gained through SARS-CoV and SARS-CoV-2 virus and vaccine modeling

### Mus musculus (Mouse)

Preclinical modeling has already provided significant insights into the mechanisms of SARS-CoV-2 infection and vaccination. Understanding the advantages and limitations of the different animal models is critical for determining relevance to the human condition, especially given species differences in not only immune biology, but also CoV2 susceptibility.

The inbred laboratory mouse continues to be the bedrock for biomedical research, particularly immune-based studies, due to cost, reagent availability, and extensive immune and genetic characterization that already exists. Mice do, however, have significant immune differences between strains that need to be taken into consideration before extrapolating to humans (29, 30). Laboratory mice are also housed under specific pathogen free conditions which severely restricts pathogen exposure and results in, for the most part, a naïve immune repertoire even as they age (31). Mice are inherently resistant to CoV2 infection due to differences between mouse vs human angiotensin converting enzyme 2 (ACE2) (mACE2 and hACE2, respectively) (32, 33). This has led to the generation and use of transgenic hACE2 and Cre-Lox induced mouse models, as well as mouse-adapted strains of CoV2 which produce robust and severe infection pathologies (34).

While mice are resistant to CoV2, they can be infected with SARS-CoV (CoV) which, while similarly utilizing a S protein that also binds ACE2, has the capacity to bind mACE2 due to differences in binding domains (35). Earlier studies with CoV showed that wild type (WT) young mice, such as BALB/c and C57BL/6, have minimal pathology following infection. However, it has been demonstrated that aged BALB/c mice experience pathology similar to that observed in severe human infection, such as pneumonia, pulmonary fibrosis, and large inflammatory cytokine responses, indicating the importance of involving age in preclinical modeling (36, 37). Vaccine efficacy, using mRNA-based vaccines to the CoV S protein, also was demonstrated in mice (38–40), with protective anti-viral antibody responses being produced. However, the mouse strain used appeared to be critical, as BALB/c mice following vaccination and then CoV viral challenge exhibited eosinophilia, lung pathology, and elevated inflammatory cytokines, events not observed in C57BL/6 (B6) mice (39, 41, 42). Another recent report supports this, as a group demonstrated that CoV2 mRNA vaccination of BALB/c mice induced significant weight loss and elevated inflammatory cytokines 1-2 days post injection, with histopathology revealing that myocarditis, as evidenced by apoptosis and necrosis, was observable as far as two weeks after initial infection, with further boosting amplifying myocarditis pathology (43). BALB/c strain mice are skewed towards producing Th2-mediated immune responses which likely contributes to these allergic-type effects, while B6 mice are more skewed towards Th1-mediated responses (44). The vaccine adjuvant was also shown to play a role, as TLR-triggering adjuvants skewing to Th1-type cytokines could ameliorate these allergic effects in the vaccinated mice, while some Th2 promoting adjuvants have been shown to promote such effects (39). A mouse adapted CoV2 has also been recently demonstrated these effects in BALB/c mice mirroring the CoV studies (45). Given that allergic reactions have been reported after vaccine administration in some people, with some only experiencing severe adverse events after boosting (46), this suggests that it is important to use multiple mouse strains to develop a more complete immunologic picture of both vaccine and infection effects to be reflective of an outbred population.

Keratin 18 (K18) hACE2 transgenic mice have the hACE2 gene put on the cytokeratin 18 promoter, allowing expression of hACE2 (47). K18 mice were originally utilized to study CoV, being used to study severe pathology that wasn’t producible in young mice. K18 mice infected with CoV and CoV2 experience pathology similar to humans, such as elevated proinflammatory cytokines and cytokine storm with severe infection, innate immune infiltrates in the lungs, and lung and systemic tissue pathology (47–51). Neuropathology typical of CoV infection is also observable in K18 mice, with neuroinvasion via the nasal and central nervous system (CNS) occurring, followed by neuronal death and activation of immune cells such as microglia and T cells, consistent with human infection responses as well (52–55). While discussed at greater detail later, K18 mice have severe pathology that limits modeling of mild or asymptomatic infection.

Commonly used mRNA vaccines targeting the CoV2 S protein have been shown to be efficacious in K18 mice by generating robust antibody responses (56–60). Similarly, IgM (61–65), IgG (26–28, 66–69), and IgA (66, 70–72) mouse antibody kinetics mirroring those observed in humans have been demonstrated. While the vast majority of these vaccine studies have centered on antibody responses, T cell responses have also been characterized (72–74). While most preclinical studies have focused on vaccine efficacy, others have demonstrated adverse effects from mRNA and S1 protein vaccination. These adverse effects include weight loss, a proinflammatory response, acute lung injury, and presence of immune infiltrates (75, 76) suggesting these models may be appropriate in delineating immune-mediated effects due solely by the vaccine.

An issue in using transgenic K18 mice concerns the overexpression hACE2 due to use of the cytokeratin 18 promoter, which is present on all epithelial tissues, and can produce pathology not typical of humans (18, 77). Overexpression of hACE2 in the nasal passages and neuroepithelium has been shown to allow for aggressive SARS-CoV-2 neuroinvasion not observed with human infection (55), while neuropathology, such as neuronal death and immune infiltrates, has been shown by others to be more severe than typically seen in humans (52–54). This overexpression also can lead to more severe organ pathology in what would be considered non-critical SARS-CoV-2 targets, such as the spleen and liver (47–51). These limitations should also be thought of when interpreting the adverse events that have been demonstrated in K18 SARS-CoV-2 vaccine models. It should also be noted that K18 mice still maintain expression of mACE2, which could further alter viral and vaccine kinetics, as well as the immune response to these challenges. While K18 mice have shown a dose dependent response to SARS-CoV-2 (78), studies are still limited and capturing nuances of human infection may prove to be a challenge in the future due to the severity of infection. While Cre-Lox and adenovirus hACE2 mouse models, which can be induced to express hACE2 on target tissues like the lungs unlike K18 mice, may be able to overcome some of the disadvantages of the K18 mouse model, these also limit mirroring of the systemic effects of CoV2 infection on tissues outside the lung (79–81).

Mouse adapted SARS-CoV and SARS-CoV-2 strains have been generated through reverse genetic engineering (82) and serial passaging in BALB/c mice (82–86). These compatible strains, such as MA10, HRB26M, MASCp6, and others, allow for the infection of young mice, particularly those of a C57BL/6 background, and tend to generate a Th1 skewed immune response with pathology similar to that seen in humans, with acute respiratory distress, lung tissue damage, pneumonia, and lung infiltrates being reported (84, 86). Both non-severe and severe infections can be modeled in a dose dependent manner, while mouse age has been shown to exacerbate pathology. Vaccine efficacy, in the form of antibody responses and resistance to the mouse adapted strains, has also been shown, using different vaccine formulations (84, 87, 88). Due to the novelty of these mouse adapted viruses, limitations have not been as extensively characterized, though it has been observed that they have a different tropism than that observed in humans, and that serially passaging induced mutations could alter viral pathology and the resulting altered immune responses (84). Further studies need to performed to establish the exact pathogenesis and immunobiology of this mouse adapted viruses, with hACE2 mouse models still primarily being used for vaccine assessment efficacy.

## Other small animal SARS-CoV-2 models *– Mesocricetus auratus* and *Mustela putorius* (Syrian hamsters and ferrets)

While most preclinical small animal CoV2 models use mice, others have had success using Syrian hamsters and ferrets due to inherent CoV2 susceptibility. Syrian hamsters offer several advantages over WT and transgenic hACE2 mouse models, with hamsters having a structurally similar ACE2 amino acid sequence and S1 protein binding site to humans (89), allowing for natural susceptibility to CoV and CoV2 infection (90–92). Because of these structural similarities, and because Syrian hamsters express ACE2 in the same tissues as humans, viral pathology is fairly similar, with the virus targeting the respiratory tract and lungs for replication, while still generating a systemic immune response (93). Viral pathology severity is dose dependent, allowing for both lethal and non-lethal SARS-CoV-2 infection, while other variables that correlate with disease severity in humans, such as age (91) and sex (94), have been observed to have similar effects on hamster outcomes following infection.

Reports on neuropathology have been conflicting, with some reporting neuronal invasion and pathology, while other have reported a lack of viral mRNA in the CNS, although neuronal immune activation and tissue damage have been consistently reported (19, 91, 95, 96). This neuropathology is more representative of human infection than what has been observed in K18 mice, and given similar viral clearance patterns to humans, hamsters have been used to model PASC (19, 96, 97). A recent report detailed structural and transcriptional changes to the lungs, kidneys, olfactory bulb, and olfactory epithelium following CoV2 infection in Syrian hamsters, with an elevated inflammatory transcriptional profile being observed in the hamsters’ brains 31 days after initial infection (19). This was accompanied by behavioral and cognitive changes. While not yet extensively performed, these models may be of particular use to also model PASC pathobiology.

While viral pathology in Syrian hamsters may more resemble humans, significant immune differences exist, although precise characterization of these differences has been hampered by limitations in validated reagents needed to delineate the complex immune responses occurring. The innate immune response is similar, with multiple groups reporting an increase in inflammatory cytokines such as TNF, IL-6, and IL-1B, as well as increase macrophage presence and activation in the lungs within the first 2-5 days of infection (91–93, 98). Adaptive immune characterization has been more difficult, however, due to limitations in hamster specific antibodies and reagents. While a robust virus specific T and B cell response has been observed, delineation and characterization of these adaptive immune responses beyond this has been limited (98). Infection also generates an antibody response similar to humans (98, 99). However, there has been acknowledgement on the limitations of hamsters in modeling human vaccine responses. Merkuleva, et al. demonstrated with a RBD-based vaccine that hamsters had a much smaller production of neutralizing antibodies in comparison to mice, rabbits, and ferrets when given the same vaccine formulation (100). Another group also demonstrated lower antibody titers in comparison to other animal models used (101), offering a potential explanation in Th2 skewing that could exist in hamsters, although this cannot be determined at this time due to the aforementioned lack of hamster specific antibodies for in-depth immune phenotyping.

Ferrets have also been extensively used in respiratory viral models, due to having a similar respiratory system to humans, as well as having similar clinical symptoms, such as coughing and sneezing (102). Ferret ACE2 is structurally similar to humans, and is also bound by the S1 protein, but actual disease severity has been shown to be mild, though severity has been shown to increase with age (not enough to be lethal however) (103–106). Neuropathology is also observable in ferrets, with viral RNA being detectable in the olfactory bulb and occipital lobe, though studies have been limited in regards to the neurological component of infection (107). PASC pathobiology has not been characterized in ferrets, and there are contrasting opinions on whether they are suitable for modeling due to the lack of disease severity (108, 109).

Studies on ferret immune responses to SARS-CoV-2 infection are limited, as, similarly to hamsters, immune-specific reagents that are ferret specific are limited. Current studies have shown the development of virus specific antibodies, with one study showing a similar response between mice and ferrets (91, 100, 110). Another group showed that with infection, a lung transcriptome profile indicating strong enrichment of genes related type 1 interferons, T cell activation, and M1 macrophage polarization, was observable, which was further elevated with age (91). Another group similarly demonstrated type 1 and type 2 interferon gene upregulation with infection, noting that this response was delayed in male ferrets (111).

## Non-human primates

Non-human primate (NPH) large animal models offer significant advantages over mouse models, being closer to humans on a genetic, physiological, immune, and even behavioral level. NHPs have similar ACE2 to humans, only differing by a few amino acids, though the amino acids that do differ are those that would be used in S1 protein binding. The major disadvantages of NHP models, in general, is the expensiveness per animal, which severely limits the numbers of animals that can be used in studies. This can also make reproducibility of data and statistics difficult due to limited animal numbers.

NHPs have pathology resembling mild human infection when inoculated with SARS-CoV-2, with clinical scoring of pneumonia, weight loss, malaise, and fever being comparable but the reduced severity of infection is problematic (112, 113). While viral replication occurs, less efficient S1 protein binding limits infectivity and pathology. One study did observed coagulation abnormalities in *Chlorocebus aethiops* (African Green Monkeys) that would be associated with more severe human infection, but this did not seem to impact long-term overall health (114). Vaccine efficacy has also been demonstrated in NHPs through antibody, immune, and antiviral responses, although studies are limited and often of a short duration, and thus adverse events have not been well documented or even assessed (115–117).

## Effects of cross-reactive secondary viral memory responses on CoV2 responses

Although the CoV2 pathogen itself may be new to our species, other human coronaviruses (hCoVs) are not and provide critical common immunological links. Seven hCoVs exist, all of which use a spike (S) protein for cellular entry. CoV2 has been linked with the original CoV due to similarities in virulence, origin, and a high molecular/genetic homology (76%) in their S-proteins (118–120). Four other hCoVs (hCoV-229E, hCoV-NL63, hCoV-HKU1, and hCoV-OC43) are endemic within the population and responsible for common seasonal minor respiratory tract infections worldwide, with the entire population generating immune responses from an early age (121–123). Significant similarities exist between CoV2 and these other hCoVs, suggesting potential cross-reactive immune responses. As with SARS-CoV and CoV2, NL63 also targets the ACE2 receptor via its S- protein. Furthermore, even stronger homologies of CoV2 exist with HKU1 and OC43, which are also beta-coronaviruses, with cross-reactive immune responses having been reported with both antibody and T cell responses (124–126). Others have already demonstrated that in addition to having similar sequence homologies, conservation of epitopes exists between SARS-CoV-2 and a few of these seasonal coronaviruses (127–129), while others have shown the potential of cross reactivity through antibody responses (124, 130, 131), which, taken together with other epidemiological evidence of cross-protective immunity (132), illustrates the potential of cross-reactive mechanisms. Immunological cross-reactivity exists not only among the hCoVs but even with coronaviruses from other species, as antibodies capable of neutralizing both human and mouse CoVs have demonstrated (133). These point to the tremendous potential of pre-existing cross-reactive secondary responses which can then affect not only resistance but also primary antigen-specific response capabilities (119). Furthermore, the endemic nature of these hCoVs indicates that continuous antigen exposure regularly occurs, further amplifying this reshaping of immune repertoire.

**Figure 1:**
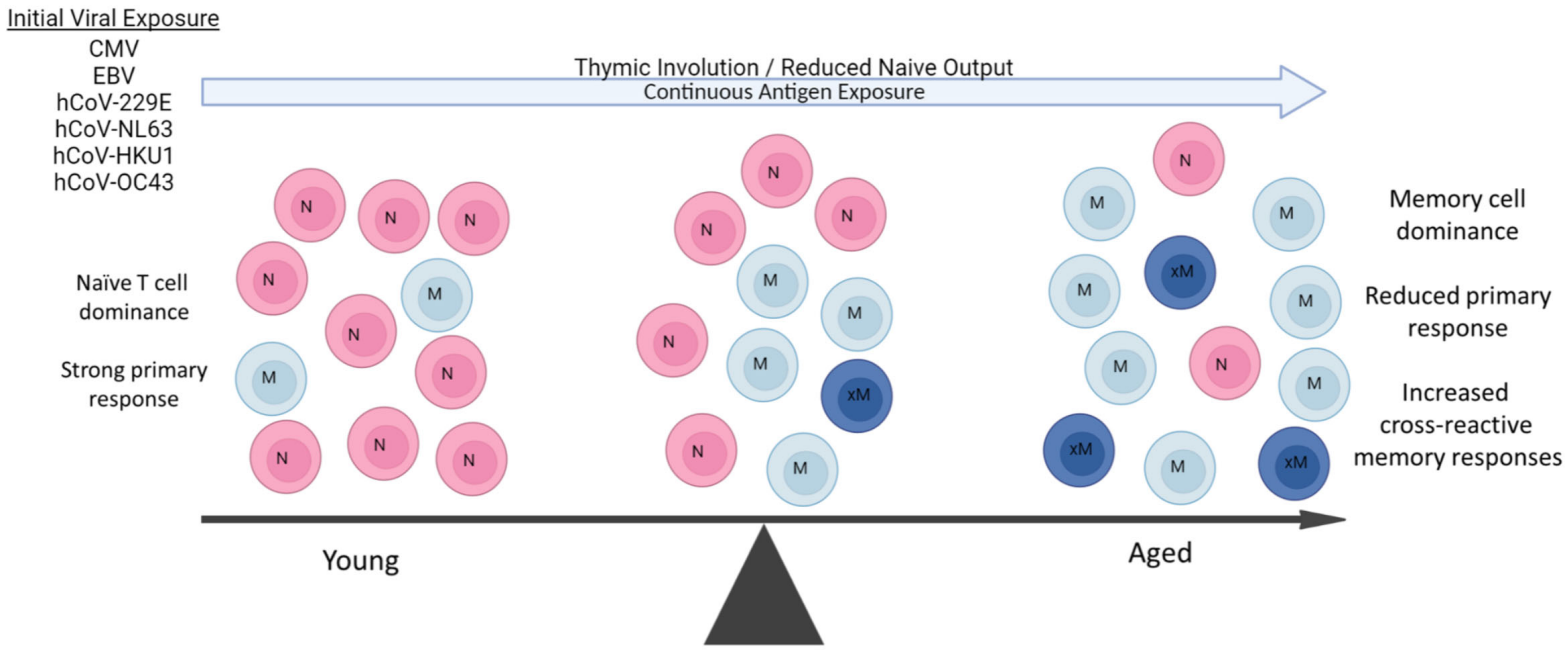
Aging Predisposes to Pre-existing Memory Responses. During aging, there is marked shift towards long-lived memory T cells due to both the massive reduction of naïve T cell output from thymic involution and constant antigenic exposure throughout life. Some viral pathogens are endemic and continuous (hCoVs), while others (CMV, EBV) are latent and a also continual source of antigen exposure. The resulting in contraction of the T cell repertoire due to naive T cell loss and predominance of memory T populations results in an increasingly impaired ability to mount primary immune responses with age. Some of these pre-existing memory cells can also be cross-reactive to new viral pathogens such as SARS-CoV-2 depending on extent of antigen similarities and affinity of original response., These cross-reactive responses can then be both of lower affinity and duration. A similar propensity for pre-existing memory B cell responses also occurs with aging, working in concert with long-lived memory CD4+ T cell help.

The tendency of the immune system to preferentially use immunologic memory from a previous infection when encountering a different version of the original stimulus has been referred to as the “Original Antigenic Sin.” This may represent a means to generate rapidly activated memory responses, even if not of high affinity, during acute infection, and provides a means to compensate in situations where optimal primary response induction is impaired. While not extensively characterized yet, the concept of original antigenic sin has been implicated in SARS-CoV-2 immunity, with recent publications supporting the impact this phenomenon could have on vaccine efficacy and disease outcome (134, 135). Other viruses, such as influenza, which have been published on more extensively in the context of antigenic sin, can potentially offer insight when extrapolated to CoV2. While cross-reactive antibody (xAb1) and T cell responses in CoV2 have been characterized, data on their overall role in efficacy of protection, either positive or negative, have been conflicting. However, many studies also illustrate the difficulty of relying on one readout especially with clinical data: use of antibody levels versus cell-mediated antigen- specific responses by T cells including an effector arm with CD8+ T cells and a helper arm with CD4+ T cells as surrogate predictors in ascertaining protection efficacy. The endemic nature of the hCoVs, with exposure occurring from a young age, combined with the presence of latent viral infections, such as CMV and EBV, add to T cell memory inflation over time, though the exact contribution of hCoVs to this memory inflation has not been well chracterized (136–139) (**Figure 1**). Preferential activation of these cross-reactive responses, even if of a lower affinity, is due to the tremendous speed advantage that memory responses have over generation of primary responses from naïve T cells. A critical question revolves around the effects of these cross-reactive pre-existing memory cells on overall efficacy towards the new pathogen, with the “net” effect highly contingent on host variables and degree of cross-reactivity. At one end, rapidly induced cross-reactive secondary immune responses, even though of lower affinity, may rapidly generate critically needed initial protection for the host and allow time for more specific primary responses to be generated, as has been suggested in CoV2 to be correlated with less severe disease (140), with a report that even prior responses to other vaccines such as to diphtheria, tetanus and pertussis (DPT), to be potential sources of protective cross-reactive responses to CoV2 (141). Another recent study demonstrated that CoV2 vaccination could induce long-lasting cross-reactive CD4^+^ T cell responses, suggesting overall better immune effects (142). Conversely, recent reports examining antibody response data from patients and mouse models demonstrated these lower affinity cross-reactive secondary responses to other hCoVs are not only less efficacious, but actually compete and inhibit primary response generation (143, 144), with suggestions that these pre-existing cross-reactive responses are deleterious in protection (145–148). These cross-reactive responses also could have a significant effect on the therapeutic application of convalescent plasma as a source of protective antibodies, which by themselves are immunogenic and also affected by anti-idiotype responses (131). How can one reconcile these potentially opposing effects being observed?

## Aging and immune responses: the increasing role of memory and cross-reactive secondary responses to overall immunity

Normal aging has been well-documented to result in significantly impaired primary antigen-specific immune responses, in part due to massive changes in the naive T-cell pool caused by thymic involution (149, 150), as well as other alterations that contribute to immunosenescence (151). Aging and obesity are also associated with a persistent pro-inflammatory state that contributes to increased naïve to memory T cell conversion (152–154). Combined with continuous pathogen exposure throughout life, this results in a marked skewing towards long-lived tissue resident memory T-cell numbers from prior antigen responses (**Figure 1**). Accordingly, the overall TCR repertoire of the aging host markedly contracts in part due to continuous expansion of long-lived memory T cells directed towards common latent viral infections such as CMV and EBV, further impacting the ability of the immune system to respond to acute viral infections (154–156). These memory T cells markedly outcompete the ever-reduced numbers of naive T cells due to their ability to rapidly respond to antigen recall, even if of lower affinity. Aging therefore increases reliance on pre-existing memory T-cell responses by the host when encountering a new pathogen. This reduced ability to mount new primary antigen-specific CD4^+^ T-cell responses would then also impair the T-cell “help” needed for the generation of high-affinity B cell and antibody generation, also favoring pre-existing cross-reactive memory B-cell responses (157–159) (**Figure 1**). Thus, at both the B and T cell levels, activated cross-reactive memory responses can potentially suppress the primary CoV2 response in both specificity and duration while at the same time initially resulting in faster responses resulting in protective effects. However, even if activated, not all of these competitive cross-reactive responses may be efficacious, given a recent report of increased production of non-protective hCoV antibodies following CoV2 infection (160). Similarly, cross-reactive hCoV-specific T cells were observed in unexposed patients (125, 126), but it was observed that after infection, the CD4^+^ T cells were weaker in response and detectable for shorter time periods (161, 162), indicative of lower functional avidity. Several of the studies also correlated the presence of these cross-reactive antibody responses with increased disease severity, suggesting a net negative effect on protection (143, 144, 163).

While notable differences in antibody responses have been noted to occur in vaccinated vs naturally infected elderly patients, age as a factor has not been rigorously assessed in studies, although there was a report assessing T cell recall responses to OC43 and NL63, which showed an absence of T cell recall responses in elderly, but not younger individuals (164), with a caveat that one cannot rule out impaired function associated with aged T cell responses. Additionally, while reports on antibody responses and cross-reactive immune responses have centered on patients following CoV2 infection (165, 166), assessment of the potential effects of vaccines on cross-reactive activation have been lacking, although one study observed no effects of a vaccine on cross-reactive antibodies versus the virus itself (167). In contrast, activation of cross-reactive S-protein-specific CD4^+^ T-cell responses following vaccination was observed, which notably did not occur in the CD8^+^ T cell population (142), but the delineation of avidity and duration of these cells versus primed naïve T cell responses was assessed. More studies are needed to determine the effects of these, and potentially other, cross-reactive immune responses following different vaccination regimens. Outside of cross-reactive T memory or antibody responses directly competing with the induction of primary responses, these pre-existing immune responses can also potentially mediate effects through activation of cross-reactive anti-idiotype responses. Understanding immune system dynamics and regulation in the context of aging could be highly revealing, especially given the potential of therapeutics already being investigated for restoring immune function and attenuating dysregulated immune responses in the elderly (168).

## Revisiting the network hypothesis: immunoregulatory effects of anti-idiotype and cross-reactive anti-idiotype responses and the role of aging

Both immunoglobulin and T-cell receptor (TCR) gene rearrangement results in the appearance of new antigenic determinants or idiotypes (Ab1 for antibody) to which the immune system has not been tolerized, which can then also induce immune responses. These anti-idiotype or Ab2 responses were postulated by Niels Jerne in the Network Theory as a means of immune-mediated regulation (169). This has been robustly demonstrated using inbred mouse models to defined antigens and monoclonal antibodies, with the bulk of research being performed in the 1980’s and 1990’s (170–173). The difficulty in demonstrating physiologically relevant Ab2 responses in humans partly stems from the tremendous heterogeneity of antigen responses in an outbred population and reliance on clinical data. Nonetheless, demonstration of anti-idiotype antibodies have been demonstrated validating the concept. The immunoregulatory effects of anti-idiotype response is due to the ability of Ab2 to bind Ab1 and neutralize it directly, or to act on the Ab1-producing B cells resulting in clearance or suppression (174–176). The cascade does not stop there however, as the Ab2 also induce down-regulatory “anti-anti-idiotype” or Ab3 responses (177). Some of the Ab3 are similar with the Ab1 idiotype response being also capable of binding the original antigen and possibly protective (178). Ab2 antibodies have even been explored to be surrogates to the original antigen as a vaccine approach. Furthermore, this Ab1>Ab2>Ab3 cascade would then allow for continuation of immune responses long after the antigen itself has disappeared which may also explain for long-lasting off-target effects of either infection or vaccination depending on the nature of the original antigen targeted.

**Figure 2:**
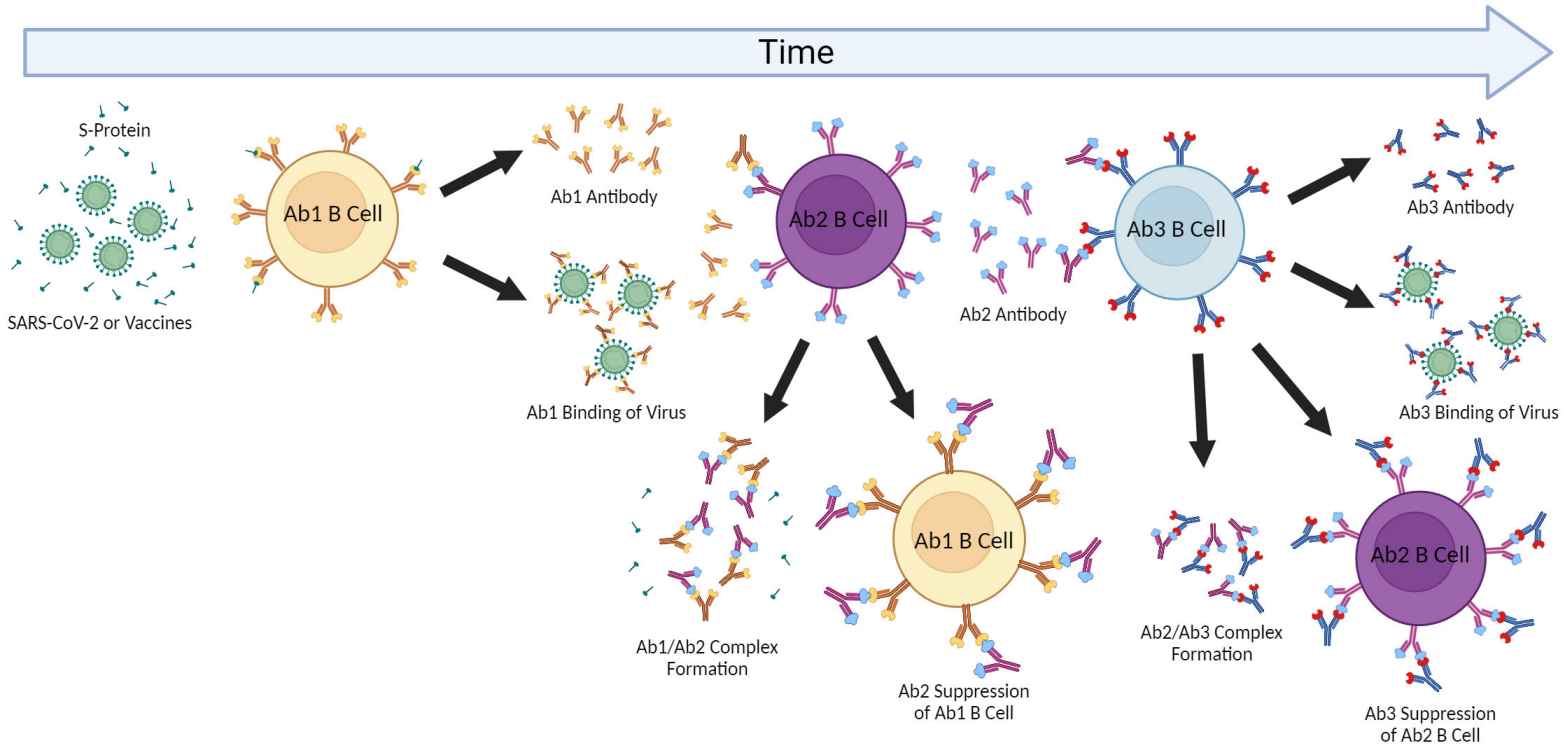
Anti-idiotype Response and the Network Cascade. The induction of antigen-specific antibody responses results in polyclonal antibodies (Ab1). The immunogenic nature of the paratope (antigen-binding region) of Ab1 then can result in polyclonal anti-idiotype (Ab2) antibodies capable of binding and inhibiting Ab1 by forming complexes resulting in Ab1 clearance. The paratope of some of these Ab2 antibodies may be a “mirror” to the original antigen and then induce anti-anti-idiotype antibody responses (Ab3) which can then regulate the down-regulatory Ab2 but also some of which may have similar binding as the Ab1 to the original antigen and be protective. This cascade and effects may be contingent on the extent of immunization or antigen exposure as repeated immunizations can result in greater Ab2 responses as well as effects on aging where cross-reactive Ab2 may also affect initial Ab1 responses.

Outside of directly affecting primary Ab1 efficacy, anti-idiotype (Ab2) responses could also potentially exert immune-mediated effects on the host which may account for longer-lasting pathologies following infection and possibly vaccination. A unique type of molecular mimicry attributed to Ab2 can mediate agonistic effects of the primary immunogen. It is important to note that Ab1 responses are polyclonal in nature, and not all Ab1 will induce the same Ab2 (which are also polyclonal), though some clones may predominate (**Figure 2**). The paratope or binding region of some, but not all, Ab2 can also represent a mirror image of the original antigenic epitope itself and as such, have the capability of binding to the cellular ligand targeted by the original antigen target. Diversity in the anti-idiotypic cascade may also partly explain the tremendous diversity of immune responses within the general population to both CoV2 infection and vaccines (179). Attempts to exploit this antigen-mimicry have included using Ab2 as surrogates for the antigen (180, 181). In the case of CoV2 or vaccines, the ACE2 receptor may be bound by Ab2 in a manner same as the CoV2 S protein (182). Detection of antibodies towards ACE2 following CoV2 infection in some patients supports this hypothesis (183). These Ab2 potentially could then mediate various off-target effects given the diverse expression of ACE2 in many tissues and cell-types, which, depending on the strength of Ab2 response, could possibly result in pathology (**Figure 2**) (182). Another receptor targeted by the CoV2 S protein, neuropilin-1 (NRP1), which is expressed in astrocytes and neurons, should also be considered in the context of Ab2 antibodies, especially given its roles in axon guidance and VEGF-A modulation. Studies have demonstrated that knockout of NRP1 can have detrimental effects, such as sympathetic nervous system dysregulated sinus bradycardia and neuronal abnormalities, such as poorly condensed ganglia and extended neurons (184). It has not yet been established if antibodies develop against NRP1 during CoV2 infection, however. Because of this, this review will focus on Ab2 responses in the context of ACE2, while noting that NRP1 should also be investigated as well.

The kinetics of Ab2 responses to both T-dependent and T-independent antigens have been extensively studied in mice, although rats and non-human primates also have been used. Initial responses to antigen demonstrated delayed Ab2 kinetics of lower magnitude (185). However, when boosting was applied, robust and rapid Ab2 responses were observed, even comparable to Ab1, while also resulting in lessening Ab1 (185). This network would then have a major effect on Ab1 efficacy and duration upon repeated stimulation. Importantly, higher Ab2 responses were also observed in older mice suggesting aging predisposes to Ab2 suppression (186). Similarly, T cells from aged mice were observed to play major role in the increased production of Ab2 at the expense of Ab1 (187). These data would suggest that cross-reactive secondary responses are induced after pathogen exposure. It is possible that cross-reactive secondary anti-idiotype (xAb2) responses may also be induced following CoV2 infection or vaccination, although this needs to be definitively shown.

**Figure 3:**
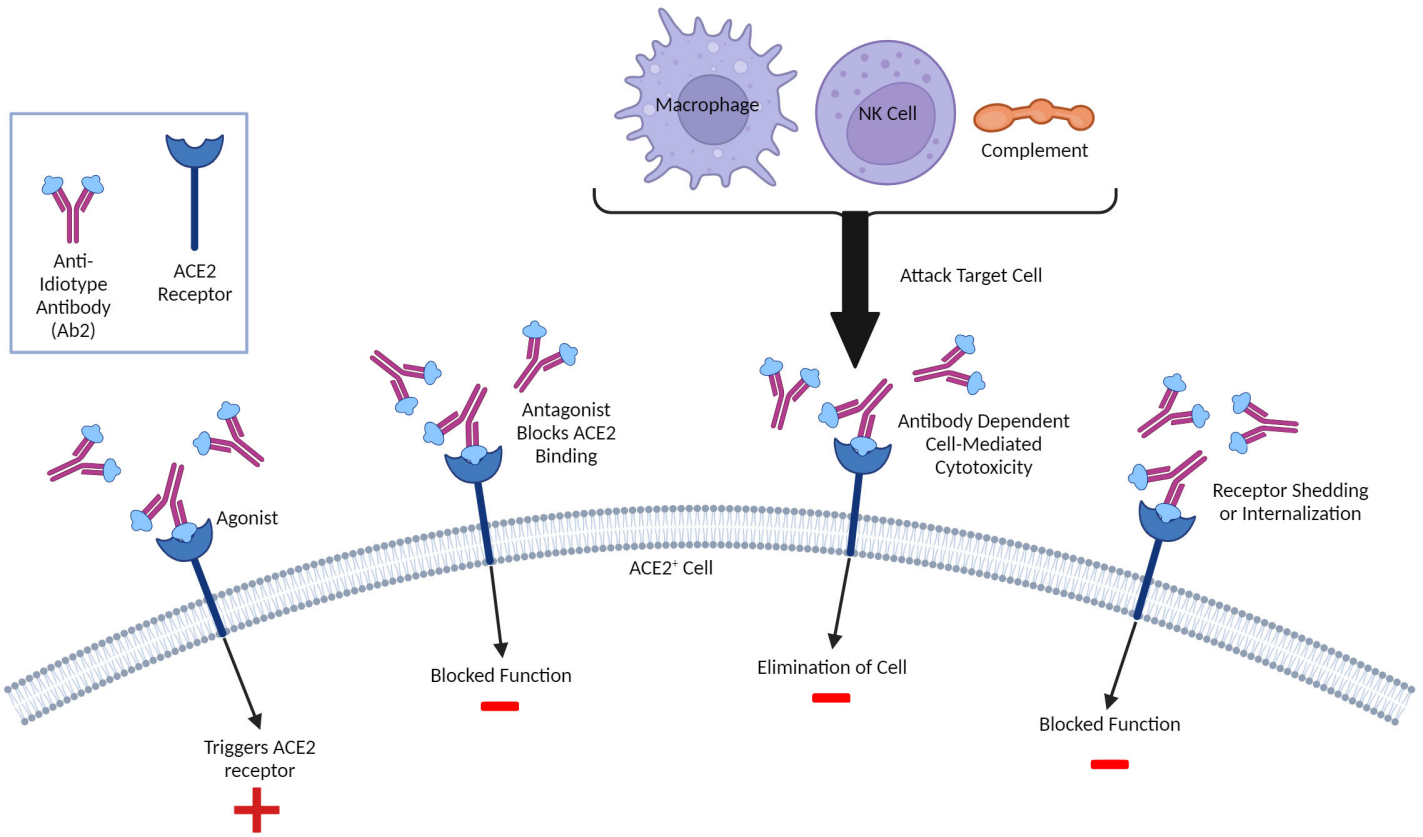
Molecular Mimicry and Potential Anti-Idiotype (Ab2)-Mediated Effects. Ab2 effects can be diverse and not limited to regulation of Ab1 responses. Ab2 are directed towards the Ab1 paratope or antigen- binding region and some can have their paratope be a mirror of the original antigen that is recognized. As such, these Ab2 can bind the same ligands the antigen which, in the case of SARS-CoV-2 infection or vaccination involving the Spike protein, can include the ACE2 receptor, resulting in multiple outcomes. If the Ab2 are antagonistic, they can competitively block ACE2 ligands from binding and inhibit function. The Ab2 upon binding can cause internalization thereby also being inhibitory. Some Ab2 could be agonistic and thereby stimulate ACE2 function. Finally, Ab2 binding ACE2+ cells can be targeted for attack by innate immune system due to ADCC (antibody-directed cell or complement-mediated cytotoxicity). A similar paradigm could exist with T cells, although this is much less characterized. Both inhibition and/or dysregulation of ACE2 function could result in potential pathology given critical role of ACE2 on multiple tissues/cells and in inflammation.

Just as cross-reactive idiotype antibodies (xAb1) or T cells can potentially directly interfere with primary immune response generation and efficacy, triggered secondary xAb2 responses, along with induced specific Ab2, could augment this down-regulatory cascade causing reduction of Ab1 responses. This may also account for the rapid waning of protective CoV2 immunity following infection or vaccination. Furthermore, repeated antigenic challenge (either by re-infection, vaccine boosting, or even continuous exposure to the original hCoV) may further amplify these inhibitory pathways (182). Aging may exacerbate these as well due to impaired primary immune response capabilities. It was reported that elderly patients following CoV2 infection had no detectable Cov2 antibodies despite having antigen-specific memory B cells, pointing to potential effects of Ab2-mediated clearance being higher in this population (188). It is also worth considering that autoantibodies to ACE2 have been previously reported in patients with connective tissue diseases (189). Antigen mimicry effects by Ab2 could also be affected by cross-reactive memory xAb2 responses based on the recognition of shared epitopes. Pre-existing xAb2 from prior NL63 infections could then also bind the ACE2 receptor and contribute to Ab2 effects. Furthermore, the continuous exposure to endemic CoVs as well as repeated administration of CoV2 vaccines could further stimulate and preferentially expand cross-reactive memory (both xAb1 and xAb2) responses, particularly with aging (**Figure 3**), possibly resulting in long-term effects that may contribute to PASC symptoms given the perpetuating cascades (Ab1, Ab2, Ab3) involved in the Network Theory. Studies are needed to ascertain if similar anti-idiotype responses are also induced after vaccinations as well as induction of comparable xAb2, which could contribute to effects given the continuous antigenic exposure to these endemic hCoVs. Determining whether increased Ab2 capable of binding ACE2 are induced following vaccination given the restricted antigen exposure versus actual CoV2 infection are also needed.

What are the implications of the anti-idiotype (either induced by Ab1 or cross-reactive) on viral protection and treatment? Other than contributing to loss of durability or down-regulation of the primary responses, they should be considered in immune-based therapies. As mentioned, therapeutic application of convalescent sera may be impacted. However, anti-idiotypic responses may have an even more profound down-regulatory effect when using monoclonal antibody-based therapeutics given the monoclonal nature of the therapeutic versus polyclonal Ab1 responses. Another issue centers on the vaccine formulation and schedule depending on the individual’s ability to mount primary immune responses. This also can be important when using vaccines targeting the original CoV2 S protein due to the continuous emergence of viral variants which result in increasing selection and loss of antigenic determinants. This extensive mutation rate of the CoV2 resulting in dominance of viral variants (Delta, Omicron, as well as the continuing emergence of new variants/subvariants), particularly within the S-protein, poses another challenge. The continuous loss of antigenic epitopes in the S-protein to which immune responses were initially generated and then expanded with current vaccine boosting could further limit efficacy due more reliance on the increasingly lower affinity cross-reactive responses. Continuous exposure to endemic hCoVs further complicates the picture by restimulating these memory responses. This has been viewed as a potential issue given the failure to generate a successful vaccine to the endemic hCoV viruses over time.

These effects may be even more pronounced in the aged population with an already impaired priming capability. One group that focused on PASC in the context of the elderly showed that an approximated 30% of patients over the age of 65 developed it (190), while another study showed that Long COVID symptoms were more severe in elderly patients (191), supporting data gathered by the US Census Bureau, though it should be noted that the elderly do not have the highest reported incidence rate of post-COVID symptoms following infection (192). Within the context of vaccination alone, the development of Long COVID has not been extensively addressed, though neuropathic symptoms have been reported, albeit at a lesser occurrence rate than primary infection without vaccination (193–195). While evidence suggests that those vaccinated before infection have protection against Long COVID (196), symptom development after vaccination alone is a phenomenon that is not understood and warrants preclinical investigation, particularly in the context of the elderly. Thus, understanding the immunology of aging in pathogen resistance is a critical parameter that needs to be incorporated into not only clinical studies but more importantly, preclinical studies, given the susceptibility of this population to not only CoV2, but other viral pathogens as well.

## Understanding the potential roles of cross-reactive secondary responses and idiotypic regulation: the importance of preclinical modeling

Fortunately, many of the issues raised are very testable hypotheses which can be mechanistically addressed using appropriate preclinical modeling. Preclinical modeling is essential for delineating the complex immunological pathways that arise following infection or vaccination at a mechanistic level, as well as ascertaining potential off-target effects over time under controlled settings. Cross-reactive immune responses, including anti-idiotype responses, on Cov2 and vaccine immunity need to be incorporated into preclinical modeling (119). It is crucial that these studies incorporate important variables such as aging, sex, obesity, and pregnancy, common conditions which all result in significant immune alterations (197–201). Immunological data involving preclinical models on CoV2 have been minimal, especially regarding long-term assessment or dissection of immune pathways under those different conditions. It is important to also recognize that while these models will provide answers, they also have significant limitations since most of these mice are housed under specific-pathogen free conditions and lack significant pathogen exposure, resulting in immature immune phenotypes. In addition, insights on off-target immune-mediated effects can also be gleaned, but choosing the appropriate model is pivotal.Long-term studies as well as effects of repeated vaccination are needed, particularly in aged mice where anti-idiotype responses may be more dominant. It will also be important to use models in the context of prior immunization involving other hCoV viral antigens and repeated stimulation or with viral challenge to allow for accumulation of memory cells. These memory cells could potentially have cross-reactive responses, impacting the ability to later mount successful primary CoV2 antigen responses, which would require a monitoring of immunoregulatory anti-idiotype responses. As anti-idiotype responses are detected after CoV2 infection directed towards ACE2 and long-term effects associated with PASC can result, it is also important to understand the potential effects these antibodies can exert in such preclinical models. Publications have already shown that the spike protein itself can cross the blood brain barrier and cause acute pathology (202–204), particularly through activation of TLR-4 and NLRP3 inflammasome associated inflammatory pathways and mechanisms (21, 205). Direct administration of S protein to the hippocampus in mice has also been shown to induce cognitive defects, behavioral abnormalities, and neuronal death, acting primarily through glial activation and the upregulation of inflammatory cytokines like IL-1B (206). While publications investigating long term pathology in preclinical models have been limited, a recent preclinical model publication demonstrated that S protein administration intracranially produced long term synaptic damage and memory impairment, accompanied by upregulation of inflammatory cytokines, such as TNF and IL-6, complement proteins, most notably C1q, and increased microgliosis, which was also previously published on in relation to spike protein activation of the NLRP3 inflammasome (21, 22). Notably, this altered neurological landscape was associated with impaired cognitive and memory function, symptoms commonly associated with PASC (22). While elucidative, other potential immune mechanisms that could be related to PASC pathology must also be assessed preclinically.

ACE2 knock-out mice have been reported to develop cardiac dysfunction and pathology as they age, with predominantly male mice being affected (207), correlating to recent reports of increased susceptibility to cardiac disease in male patients recovering from CoV2 infection (10). Some tissues from ACE2 knock-out mice also can display increased inflammatory responses during certain stimuli (208) indicating that preclinical assessments should involve various immunostimulatory challenges in CoV2 models, although confounding issues may arise due to inappropriate expression of hACE2 in the tissues of these mice (209), as well as competition with mouse ACE2. Preclinical modeling using multiple approaches and conditions are needed, and it is crucial to take into consideration the limitations of each model before attempting to extrapolate results to the general population.

**Figure 4:**
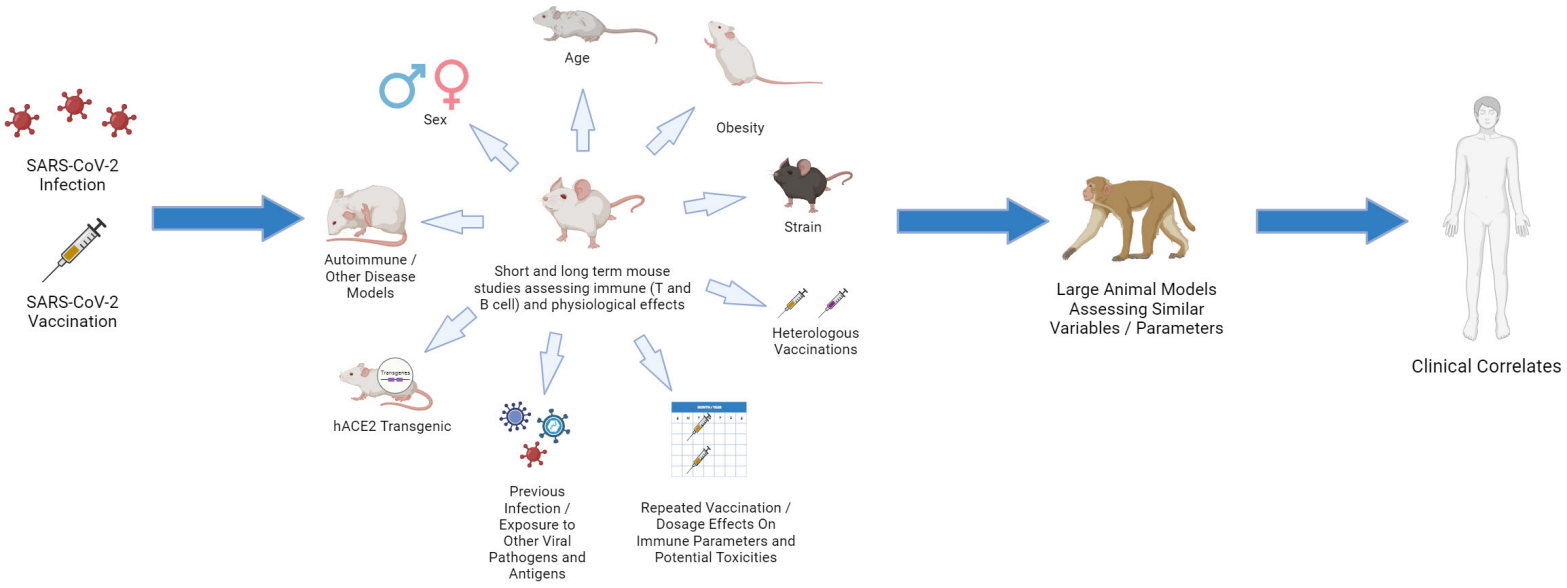
Choosing the Relevant Preclinical Models for CoV2: “Bench to Bedside Back to Bench”. Using young, inbred laboratory mice under SPF conditions fails to mirror the human landscape. Use of aged and obese mice, different strains, pregnant mice, mice that are prone to various autoimmune and disease states, and mice that have been exposed to various pathogens (including HCoV antigens) can all provide important insights. Studies in which repeated vaccinations, including heterologous, are applied and dissection of immune responses, including in different tissues, are assessed. These preclinical studies can then be linked with large animal models, such as non-human primate (NHP) using similar parameters as aging and obesity, which more faithfully represent human immune dynamics. The data can then be linked and validated with clinical results which then can drive questions using the various preclinical models on immune response efficacy and maintenance.

Preclinical studies on CoV2 responses are urgently needed to answer questions about potential effects of prior cross-reactive immunity on primary response generation and duration, tracking not only anti-idiotype responses but also other immunoregulatory pathways over time, as well as effects of heterologous and repeated vaccinations (**Figure 4**). It would also be pertinent to investigate these mechanisms in the context of viral infection as well, which is especially relevant given high reinfection rates reported with SARS-CoV-2. Such experiments would also allow for assessment of potential short- and long-term immune-mediated off-target effects on different tissues, a particularly pressing need given the complex biology and effects of ACE2 and other molecules by which the CoV2 S-protein can bind. The use of antibodies to ACE2 in mice as well as more complete characterization of the role of ACE2 on physiologic functions in various tissues may shed some light on issues surrounding PASC which is especially important given increased cardiac risks associated with infection after resolution.

Therefore, increasing basic science investigation on the potential effects of pre-existing immune responses underlying prior viral infections and their potential impact on CoV2, as well as vaccine immune responses in the context of aging, are critical next steps in the continuing fight against the current, and possibly future, viral pandemics. Moving forward, given the significant gaps in knowledge on the effects of both the virus and associated vaccines on immune responses and effects, preclinical studies should emphasize human modifying variables such as obesity and aging combined with repeated stimulation on immune responses and possible off-target effects. It is only in preclinical models that control for exposure to the stimulus (virus or antigen) and incorporating these different variables can result in definitive data. The data observed with the original SARS-CoV vaccines and infection also point to potentially immunopathologic responses which were augmented in aged mice and in some cases, strain-dependent. Thus, it is perhaps not surprising that similar effects may occur with CoV2 and vaccines which need to be further delineated. The models should also be developed to better model PASC and the potential role of immune responses or the virus and associated antigens in perpetuating it these long-term effects. As the CoV2 virus continually changes, so do the vaccine formulations to combat it, and thus these variables also need to be incorporated in the preclinical models. Basic studies are still needed delineating the amount of protein being transcribed, by what cells and for how long as well as what variables affect it. The different components of the vaccine product need to also be more stringently studies regarding immune effects. In the case of the mRNA CoV2 vaccines, this involves understanding the immunogenicity of the NLP carrier, the mRNA itself, and finally the S protein made, both individually and as a composite on immune responses. Regarding anti-idiotype responses, studies in which transfer of anti-S antibodies to mice and assessing both longevity and generation of anti-idiotype responses will be revealing. This includes not only effects on adaptive (T and B cell) but also innate immune components. Finally, different dosing regimens need to be better assessed in determining boosting strategies also keeping in mind critical aspects such as age given the significant differences in immune status of young, adult, and advanced aged recipients towards any pathogen or antigenic challenge.

## Author contributions

CC: Writing – original draft, Writing – review & editing. DL: Writing – original draft, Writing – review & editing. WM: Writing – original draft, Writing – review & editing.

## Glossary

**Table:**

**Idiotype:** The antigenic part of the specific antigen-binding domain within the variable region of an immunoglobulin (Ab1) or TCR. With most antigens, these are polyclonal responses consisting of multiple idiotypes of different affinities.
**Paratope:** The region of an antibody that binds a specific antigen or epitope.
**Anti-idiotype:** Antibody or T-cell responses directed to idiotypes due to immunogenicity by being novel proteins arising from gene rearrangements of immunoglobulin or TCR. Anti-idiotype (Ab2) responses are also polyclonal and may not be induced to all Ab1 or to the same extent. Some Ab2 paratope regions may resemble the original antigen as a form of molecular mimicry and are capable of inducing immune responses similar to the antigen itself. Some of these Ab2 are therefore also of binding the ligand of the original antigen.
**Anti-anti-idiotype:** Immune responses (Ab3) targeting the idiotype of anti-idiotype (Ab2). Ab3 may resemble the Ab1 in specificity due to some Ab2 paratopes mirroring the original antigen.
**hCoV:** Human coronaviruses known for their characteristic spike (S) protein which facilitates entry into the target cell. There are seven hCoVs: hCoV-229E, hCoV-NL63, hCoV-OC43, hCoV-HKU1, MERS-CoV, SARS-CoV, and SARS-CoV-2 of which 229E, NL63, OC43, and HKU1 are endemic.
**Original antigenic Sin:** A phrase used to describe a phenomenon in which prior immune responses to other antigens/epitopes can cross-react (xAb1) and bind to a new antigen and possibly interfere with primary immune responses (Ab1) to the new antigen. These can also involve anti-idiotype (xAb2) responses.
**Network hypothesis:** A theory proposed by N Jerne to explain immunoregulatory pathways governing antigen-specific B and T cell responses over time in which immune responses are generated to initial adaptive immune cell products as a form of down-regulation.
**Outstanding questions** Are cross-reactive memory responses beneficial or deleterious in CoV2 protection and does age or obesity influence these effects? What are the differences between CoV2 infection versus vaccine application on activation of cross- reactive and anti-idiotype responses? Do cross-reactive anti-idiotype responses occur after infection or vaccination and are anti-idiotype responses increased in aging playing a role in the diminution of protective responses or off-target or long-term pathology? What preclinical model conditions can best mirror the human scenario and be used to assess potential roles of cross-reactive memory responses and anti-idiotype effects? What vaccine regimens and types are needed in different clinical populations (age, obesity, comorbid illness as cofactors) to increase immune efficacy and maintain duration considering the CoV2 variants the arise?
**Significance:** Cross-reactive memory idiotype and anti-idiotype responses from prior pathogens may contribute and possibly interfere with the generation of primary antigen-specific responses to both SARS-CoV-2 infections and associated vaccines. Due to the predominance of memory T-cell and potentially anti-idiotypic responses, these effects may be heightened in aging and may also contribute to off-target effects. Preclinical modeling incorporating these variables are needed to follow immune responses over time and determine optimal regimens for vaccine application as well as potential therapeutic strategies to increase immune efficacy and durability.
